# A rare isolated unilateral myositis ossificans traumatica of the lateral pterygoid muscle: a case report

**DOI:** 10.1186/1752-1947-8-230

**Published:** 2014-06-26

**Authors:** Alessia Spinzia, Guido Moscato, Emanuele Broccardo, Lara Castelletti, Fabio Maglitto, Giovanni Dell’Aversana Orabona, Pasquale Piombino

**Affiliations:** 1 Unit of Odontostomatology and Maxillofacial Surgery, IRCCS San Martino - IST University Hospital, Largo R. Benzi 10, 16132 Genoa, Italy; 2 Unit of Neuroradiology, IRCCS San Martino - IST University Hospital, Largo R. Benzi 10, 16132 Genoa, Italy; 3 MaxilloFacial Surgery Unit, Department of Neurosciences and Reproductive and Odontostomatological Sciences, University of Naples ‘Federico II’, via Pansini, 5, 80131 Naples, Italy

**Keywords:** Myositis ossificans traumatica, Lateral pterygoid muscle, Physiotherapy treatment, Temporo mandibular joint disorders

## Abstract

**Introduction:**

Myositis ossificans traumatica is a pathological condition characterized by the extraskeletal formation of bony tissue, induced by violent or repeated trauma.

**Case presentation:**

A 30-year-old Italian man, after surgical treatment for multiple facial fractures, presented with a progressive limitation of mouth opening. A computed tomography scan showed a significant calcification of the fibers of the left lateral pterygoid muscle. The working diagnosis was myositis ossificans traumatica of the left lateral pterygoid muscle. Surgical excision was suggested but not performed. Our patient underwent physiotherapy treatment resulting not in a complete restoration of mandibular movements but in an acceptable recovery of mouth opening.

**Conclusions:**

Myositis ossificans is a rare complication that can be caused by muscle trauma. Therefore, special attention should be paid to surgical trauma. In the present case, surgical excision was considered, in accordance with the literature, and suggested to our patient, but he declined due to the absence of any pain or any significant limitation to his daily life activities. He therefore underwent physiotherapy treatment, in line with our unit’s guidelines, resulting not in a complete restoration of mandibular movements but in an acceptable recovery of mouth opening.

## Introduction

Myositis ossificans (MO) is a relatively rare disease characterized by the formation of mature bone in extraskeletal sites [1]. Myositis ossificans can be divided into two groups: progressive MO and traumatic MO.

Progressive myositis ossificans or Munchmeyer’s disease is a hereditary form with autosomal dominant transmission. It causes symptoms from early infancy and involves several muscles. The consequent functional limitations are progressive and disabling. Normally, there are associated skeletal malformations, disorders of sexual development and deafness [1]. Involvement of the head and neck can also occur in 20 percent of patients [2].

Myositis ossificans traumatic (MOT) is a more circumscribed form, which involves single muscles or muscle groups subjected to violent or repeated trauma [1].

MOT is also called myositis ossificans circumscripta. It is frequently reported in orthopedic literature, often involving the quadriceps femoris and brachialis anticus, and seems to occur after significant blunt trauma, such as a sport-related injury or repeated minor injuries. However, the maxillofacial district is seldom involved, with only 52 known cases reported [2].

We report a case of isolated unilateral MOT of the left lateral pterygoid muscle.

## Case presentation

A 30-year-old Italian man was referred in June 2010 to our unit for multiple facial fractures resulting from a motorcycle accident.

**Figure 1:**
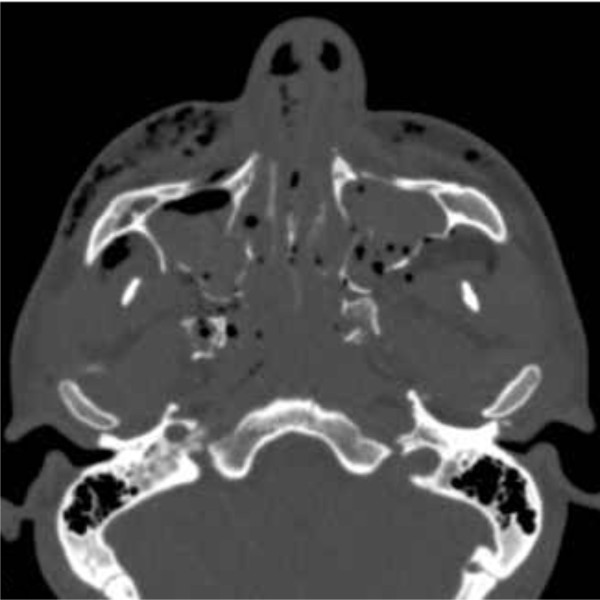
Pre-operative computed tomography scan in axial projection demonstrating bilateral pterygoid process fracture.

A physical examination demonstrated a diffuse edema in the middle and lower facial thirds, bilateral periorbital edema and diffuse escoriation to his cheeks. On intraoral examination severe preternatural mobility of the upper and lower jaws was noticed, with malocclusion, anterior open bite and mucosal lacerations. Our patient also complained of bilateral numbness to the nasolabial region and cheek.A computed tomography (CT) scan revealed fractures to the nasal bones, associated 1, 2 and 3 Le Fort fractures, right paramedian lower jaw, bilateral zygomatic fractures, orbital floor, sphenoidal sinus wall and pterygoid processes (Figure 1).

Our patient underwent surgery under general anesthesia. An oral, bilateral transconjunctival and transcutaneous approach was performed for the reduction and fixation of the major facial fractures. The surgical treatment did not require any intermaxillary fixation.

**Figure 2:**
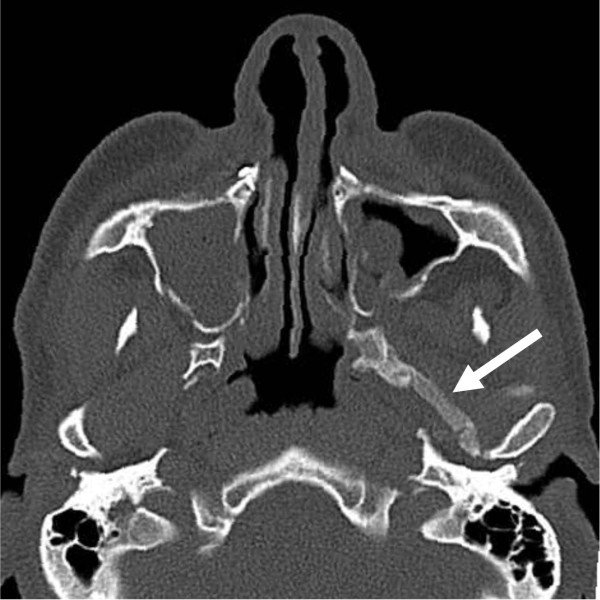
Postoperative computed tomography scan in axial projection one month after surgery demonstrating significant calcification of fibers of the left lateral pterygoid muscle (arrow).

Our patient was submitted to clinical and radiological postoperative follow-up. One month later, in July, our patient complained of the onset of a gradual limitation in mouth opening. His maximal incisal opening (MIO) was 10mm with an almost complete absence of any protrusive, right and left lateral mandibular movements (Table 1).Based on our patient’s symptoms, a CT scan was ordered a few days later, which showed a significant calcification of the left lateral pterygoid muscle. An irregular ossified mass extending from the left pterygoid process to the mandibular condyle was noticed (Figure 2). The working diagnosis was MOT of the left lateral pterygoid muscle.

**Table 1:** Mandibular movement values during follow-up

**Clinical follow-up**	**MIO (mm)**	**Protrusive (mm)**	**Right laterality (mm)**	**Left laterality (mm)**
One month after surgery	10	0	0	0
After 1 month of FKT	25	0.5	0.5	2
After 12 months of FKT	30	1	1	3

MIO: maximal incisal opening; FKT: physiotherapy.

We suggested a surgical removal of the foci of ossification, but our patient refused due to the absence of any pain or any significant limitation in the activities of his daily life. Therefore, he was immediately advised to undergo physiotherapy (FKT), in accordance with our unit’s guidelines.

A set of exercises consisting of forced active and passive mouth opening was presented to our patient. The training was aimed to correct his jaw’s movable model to reach a satisfactory range of movements.

Our patient was instructed to open his mouth in a straight line, in front of a mirror. He then had to practice protrusion and left and right lateralization movements. Our patient accomplished each movement by applying gentle pressure with his fingers. The exercises had to be performed four times a day in batches of about 20 repetitions for every vector of motion.

He returned 30 days later, with a MIO of 25mm and a minimal improvement of mandibular movements (Table 1).

**Figure 3:**
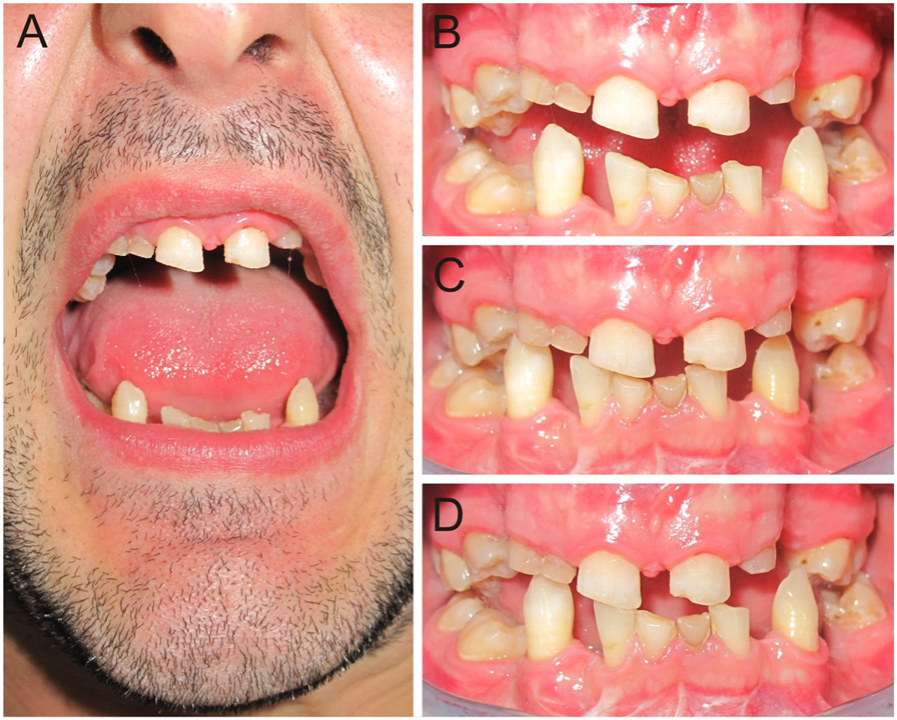
**Post-physiotherapy (12 months) mandibular movements. (A)** Maximal incisal opening; **(B)** protrusion; **(C)** right laterality; **(D)** left laterality.

**Figure 4:**
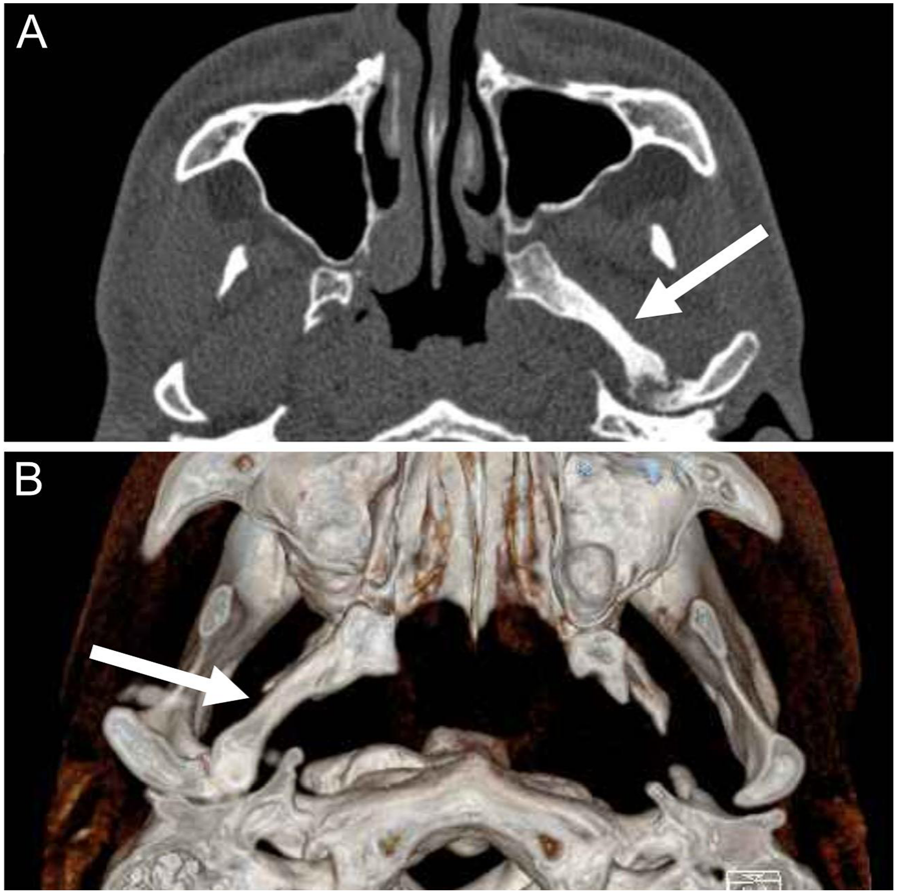
**Post-physiotherapy (12 months) computed tomography scan. (A)** Axial projection, upper arrow pointing the calcifications of the left lateral pterygoid muscle; **(B)** three-dimensional volume rendering, lower arrow pointing the calcifications of the left lateral pterygoid muscle (upper view).

In July 2011, 12 months after starting physiotherapy, our patient had improved the range of motion of the mandible, reaching a MIO of 30mm (Table 1) without any significant restoration of other mandibular movements (Figure 3). Furthermore, another CT scan revealed the persistence of calcifications of the left lateral pterygoid muscle (Figure 4) explaining the incomplete resolution of the clinical picture.

## Discussion

MOT involving the lateral pterygoid muscle is rare with only 10 cases reported in the literature [3-11] including the present case (Table 2). There is a male to female predominance of 7:3, the average age of the patients is 36.6 years (range 21 to 65 years).

**Table 2:** Case reports in the literature of myositis ossificans traumatic involving the lateral pterygoid muscle

**Author**	**Year**	**Gender**	**Age**	**Location**	**History of trauma**	**Treatment**	**Recurrence**
Abdin and Prabhu [3]	1984	F	43	Lateral pterygoid	Y	Surgical	N
Lello and Makek [9]	1986	M	34	Temporal, lateral pterygoid	Y	Surgical	N
Spinazze *et al*. [11]	1998	M	55	Masseter, temporal, lateral pterygoid, medial pterygoid	Y	Surgical	Y
Aoki *et al*. [4]	2002	M	44	Masseter, lateral pterygoid, medial pterygoid	Y	Surgical	Y
Kim *et al*. [8]	2002	F	30	Lateral pterygoid, sternocleidomastoid	N	Surgical	Y
Godhi *et al*. [6]	2011	M	21	Lateral pterygoid, temporal	N	Surgical	Y
Ebbert *et al*. [5]	2012	M	45	Lateral pterygoid, medial pterygoid	N	Not declared	U
Nemoto *et al*. [10]	2012	M	39	Frontal, temporal, lateral pterygoid, masseter	Y	Surgical	N
Jayade *et al*. [7]	2013	F	25	Lateral pterygoid, medial pterygoid, temporal	N	Surgical	N
Present case	2013	M	30	Lateral pterygoid	Y	Physical therapy	N

F: female; M: male; Y: yes; N: no; U: unknown.

In most cases the lateral pterygoid is involved jointly with other muscles of the maxillofacial district. Only one other case [3] and our case describe isolated unilateral MOT of the lateral pterygoid muscle.

MOT describes a reparative process whereby benign heterotopic ossification occurs in the soft tissues [12].

Concerning the etiology of MOT of the masticatory muscles, several causes have been described: tooth extraction, migrating odontogenic abscess, local anesthetic injection, cervical collar, genioplasty, badly performed orthodontic treatment, direct force and facial skeleton fractures [13].

There are many theories about the pathogenesis of MOT and little agreement about the exact mechanism involved [1].

Carey [14] proposed four main theories for its development: the displacement of bony fragments into the soft tissue with subsequent proliferation; the detachment of periosteal fragments into the surrounding tissue with a proliferation of the osteoprogenitor cells; the migration of subperiosteal osteoprogenitor cells into the surrounding soft tissue, through a periosteal perforation induced by trauma; and the metaplasy of extraosseous cells exposed to bone morphogenetic proteins (BMP) derived from the lysis of bone fragments displaced within the soft tissue during traumatic injury. Arima *et al*[15] proposed that the autolysis of scattered bone fragments releases bone morphogenetic protein, inducing the differentiation of the perivascular mesenchymal cells into the muscular tissue, resulting in a relatively homogenous bony mass. They also found that the interval between the trauma and first detection of a calcified mass ranged from three weeks to more than 20 years.

The most accredited theory, mentioned in many scientific articles [3,12,16], suggests that MOT results from an initial intramuscular hemorrhage with an exuberant proliferation of vascular granulation tissue that undergoes subsequent metaplasy to the cartilaginous bone.

Males are three times more prone to trauma than are women, because of their more active lifestyle. The region where MOT most frequently occurs is the masseter muscle, which may be attributed to the fact that the masseter muscle is on the outside of the mandible and is likely to receive external force directly [3]. But cases involving the medial pterygoid, temporal, sternocleidomastoid, mental, platysma, scalenus, omohyoid, buccinator and genioglossus muscles have also been reported. In contrast, the lateral pterygoid muscle is rarely affected, with only one case of isolated unilateral MOT reported in the literature, probably caused by a severe form of facial cellulitis [3].

In our case, our patient had a clear history of facial trauma with a pterygoid process fracture probably leading to MOT of the left lateral pterygoid muscle.

Patients with MOT involving the muscles of mastication typically present with a limitation of MIO.

The real challenge is the differential diagnosis between MOT and other diseases that can result in a reduced mouth opening. Both intra-articular (ankylosis, the anchored disc phenomenon, and bilateral anterior disc displacement without reduction) and extra-articular (infection, foreign body reaction, musculoskeletal injury or disorder, enlargement of the coronoid process and neoplasm) causes must be considered in the diagnostic hypotheses. A clinical suspicion of MOT must be confirmed and supported by radiological findings.

A CT scan is useful both for the diagnosis and planning of surgical treatment, being able to identify the exact location and relationship of the lesion with the surrounding tissues. The bony mass shows a zonal pattern characterized by multiple foci of central noncalcified regions of low attenuation, surrounded by a peripheral ring of high density, consistent with mature bone.

Different treatment strategies have been discussed in the literature for MOT: surgical treatment (excision), physiotherapy (including TheraBite™), medical therapy (nonsteroidal anti-inflammatory drugs, bisphosphonates, magnesium, warfarin) and low-dose radiation therapy [12]. There is no general consensus concerning the effectiveness of nonsurgical therapy for MOT, and this requires further research.

The only treatment modality accepted universally is the complete excision of the ossified mass as early as possible. This should be followed by aggressive postoperative physiotherapy.

Other authors recommend the avoidance of surgical intervention unless a functional disability develops or the lesion regresses, claiming that 35 percent of MOT cases will spontaneously resolve over several months [17].

## Conclusions

The initial management of myositis ossificans depends on the stage of development. The surgical excision is the recommended treatment if the patient has pain or restricted motion. Excision is considered when the lesion reaches maturity, usually at six to 12 months to avoid recurrence. In the present case, in accordance with the literature, surgical excision was considered and suggested to our patient. But he declined due to the absence of any pain or any significant limitation in his daily life activities. Therefore, he underwent physiotherapy treatment, in line with our unit’s guidelines resulting not in a complete restoration of mandibular movements but in an acceptable recovery of mouth opening.

## Consent

Written informed consent was obtained from the patient for publication of this case report and any accompanying images. A copy of the written consent is available for review by the Editor-in-Chief of this journal.

## Abbreviations

BMP: bone morphogenetic protein; CT: computed tomography; FKT: physiotherapy; MIO: maximal incisal opening; MO: myositis ossificans; MOT: myositis ossificans traumatica.

## Competing interests

The authors declare that they have no competing interests.

## Authors’ contributions

PP was the major contributor in writing the manuscript. FM reviewed the manuscript. GDO and GM were responsible for the clinical management of the patient. LC performed the radiology analysis. AS and EB performed the surgery. All authors read and approved the final manuscript.

## References

[B1] Mevio E Rizzi L Bernasconi G Myositis ossificans traumatica of the temporal muscle: a case report Auris Nasus Larynx 2001 28 345 347 10.1016/s0385-8146(01)00059-1 11694380

[B2] Ramieri V Bianca C Arangio P Cascone P Myositis ossificans of the medial pterygoid muscle J Craniofac Surg 2010 21 1202 1204 10.1097/SCS.0b013e3181e17cfa 20613616

[B3] Abdin HA Prabhu SR Traumatic myositis ossificans of lateral pterygoid muscle J Oral Med 1984 39 54 56 6585498

[B4] Aoki T Naito H Ota Y Shiiki K Myositis ossificans traumatica of the masticatory muscles: review of the literature and report of a case J Oral Maxillofac Surg 2002 60 1083 1088 10.1053/joms.2002.34427 12216001

[B5] Ebbert TL Baima JJ Jr Smoker WR Myositis ossificans of the bilateral medial and lateral pterygoid muscles Arch Otolaryngol Head Neck Surg 2012 138 422 423 10.1001/archoto.2012.62a 22508629

[B6] Godhi SS Singh A Kukreja P Singh V Myositis ossificans circumscripta involving bilateral masticatory muscles J Craniofac Surg 2011 22 e11 e13 10.1097/SCS.0b013e31822ec7cc 22134307

[B7] Jayade B Adirajaiah S Vadera H Kundalaswamy G Sattur AP Kalkur C Myositis ossificans in medial, lateral pterygoid, and contralateral temporalis muscles: a rare case report Oral Surg Oral Med Oral Pathol Oral Radiol 2013 116 e261 e266 10.1016/j.oooo.2011.11.036 22819452

[B8] Kim DD Lazow SK Har-El G Berger JR Myositis ossificans traumatica of masticatory musculature: a case report and literature review J Oral Maxillofac Surg 2002 60 1072 1076 10.1053/joms.2002.34424 12215998

[B9] Lello GE Makek M Traumatic myositis ossificans in masticatory muscles J Maxillofac Surg 1986 14 231 237 10.1016/s0301-0503(86)80295-8 3461100

[B10] Nemoto H Sumiya N Ito Y Kimura N Akizuki A Maruyama N Myositis ossificans traumatica of the masticatory muscles J Craniofac Surg 2012 23 e514 e516 10.1097/SCS.0b013e31825b33de 22976727

[B11] Spinazze RP Heffez LB Bays RA Chronic, progressive limitation of mouth opening J Oral Maxillofac Surg 1998 56 1178 1786 10.1016/s0278-2391(98)90767-4 9766544

[B12] Thangavelu A Vaidhyanathan A Narendar R Myositis ossificans traumatica of the medial pterygoid Int J Oral Maxillofac Surg 2011 40 545 549 10.1016/j.ijom.2010.10.024 21112739

[B13] Ungari C Filiaci F Riccardi E Rinna C Iannetti G Etiology and incidence of zygomatic fracture: a retrospective study related to a series of 642 patients Eur Rev Med Pharmacol Sci 2012 16 1559 1562 23111970

[B14] Carey EJ Multiple bilateral parosteal bone and callus formations of the femur and left innominate bone Arch Surg 1924 8 592 603

[B15] Arima R Shiba R Hayashi T Traumatic myositis ossificans in the masseter muscle J Oral Maxillofac Surg 1984 42 521 526 10.1016/0278-2391(84)90011-9 6588169

[B16] Ungari C Quarato D Gennaro P Riccardi E Agrillo A Mitro V Cascino F Reale G Rinna C Filiaci F A retrospective analysis of the headache associated with temporomandibular joint disorder Eur Rev Med Pharmacol Sci 2012 16 1878 1881 23208975

[B17] Conner GA Duffy M Myositis ossificans: a case report of multiple recurrences following third molar extractions and review of the literature J Oral Maxillofac Surg 2009 67 920 926 10.1016/j.joms.2008.06.106 19304059

